# Esophagitis Dissecans Superficialis (EDS) Secondary to Hair Dye Ingestion: Case Report and Literature Review

**DOI:** 10.3390/clinpract11020026

**Published:** 2021-03-29

**Authors:** Eric Omar Then, Tyler Grantham, Michell Lopez, Madhavi Reddy, Vinaya Gaduputi

**Affiliations:** 1 Division of Gastroenterology and Hepatology, The Brooklyn Hospital Center, Clinical Affiliate of The Mount Sinai Hospital, Brooklyn, NY 11201, USA; Mreddy@tbh.org; 2 Division of Gastroenterology and Hepatology, SBH Health System, Bronx, NY 10457, USA; Mlopez@sbhny.org (M.L.); Vgaduputi@sbhny.org (V.G.); 3 Department of Internal Medicine, St. George’s University School of Medicine, West Indies, Grenada; tgrantha@sgu.edu

**Keywords:** esophagogastroduodenoscopy, esophagitis dissecans superficialis, esophagitis

## Abstract

Esophagitis dissecans superficialis (EDS) is a rare and underdiagnosed esophageal lesion characterized by sloughing of the esophageal mucosa that has been associated with medications, various autoimmune disorders, and exposure to some chemical irritants. Anatomically, EDS is most commonly seen in the middle and distal thirds of the esophagus. When present, EDS is best treated by discontinuing the offending agent and initiating pharmacologic therapy with proton pump inhibitors. Steroids may also be effective if the etiology is autoimmune in nature. Our case highlights a 65-year-old female diagnosed with EDS after incidental ingestion of hair dye containing resorcinol and para-phenylenediamine (PPD).

## 1. Introduction

Esophagitis dissecans superficialis (EDS) is a rare and underdiagnosed esophageal lesion characterized by sloughing of the esophageal mucosa. This is primarily an endoscopic finding as histologic features are often nonspecific and difficult to define [1]. Despite its alarming appearance, EDS is typically benign and limited in nature. There are rarely severe complications and most patients do not have features of sloughing upon follow-up [2]. The pathogenesis is unclear and is often considered to be idiopathic. However, various etiologies have been discussed in the literature. Most notably, this has been seen with autoimmune disorders and with exposure to certain medications. It has also been thought to be associated with iatrogenic esophageal injuries and exposure to chemical irritants. Our case highlights a 65-year-old woman diagnosed with EDS after incidental ingestion of hair dye.

## 2. Case Report

**Figure 1:**
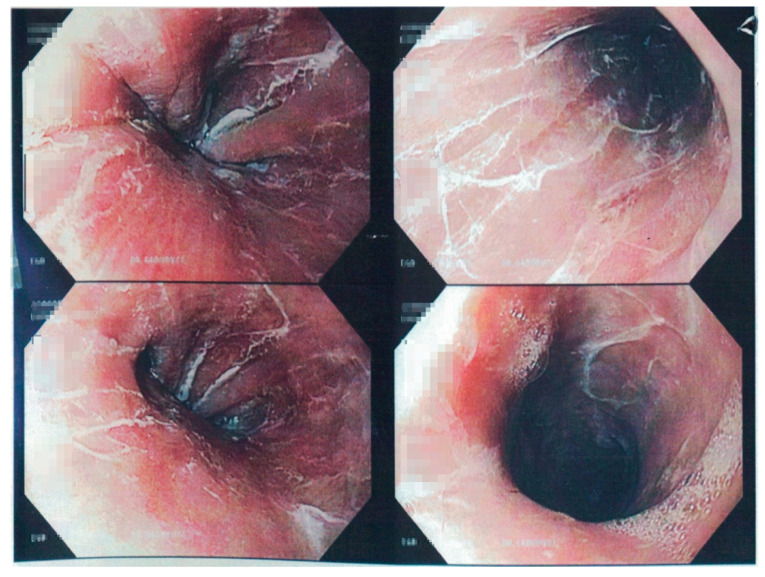
Esophagogastroduodenoscopy (EGD) showing superficial mucosal desquamation in entire esophagus suggestive of esophagitis dissecans superficialis.

The patient is a 65-year-old female with a medical history of diabetes mellitus type 2, hypertension, major depressive disorder, asthma, alcohol use disorder who presented to our emergency department complaining of nausea, vomiting, odynophagia, and throat pain after ingesting a colored hair dye. The patient stated she accidentally ingested 2 ounces of hair dye believing it was cough syrup. On medication review, the patient was taking sertraline, albuterol, acetaminophen, metformin, and N\nifedipine as an outpatient. Notable laboratory findings on admission included a complete blood count within normal limits; a venous blood gas showing a pH of 7.274, pCO_2_ of 39.2 mmHg, HCO_3_ of 17.2 mEq/L, and lactic acid of 5.1. Notable imaging included a chest X-ray showing focal patchy opacities at both lung bases. An X-ray of the neck was also acquired and showed no retropharyngeal soft tissue swelling. On physical exam, the patient demonstrated dysphonia but was otherwise unremarkable. After initial evaluation, the patient was placed nil per os (NPO), and the gastroenterology service was consulted for further recommendations. An esophagogastroduodenoscopy (EGD) was promptly performed and showed mild superficial mucosal desquamation in the entire esophagus consistent with esophagitis dissecans superficialis (Figure 1). After the procedure, the patient was started on intravenous pantoprazole and admitted to the medical ward for further monitoring. The following day, the patient’s diet was advanced to clear liquids, and later that night, she was able tolerate a solid diet. Subsequently the patient’s symptoms were resolved, and she was safely discharged home with follow-up appointments scheduled. On follow-up appointment, the patient reported continued improvement, and as such, refused to undergo repeat EGD to evaluate for mucosal healing.

## 3. Discussion

EDS was first described in 1892 and has since been widely under-recognized. One retrospective study of 21,497 upper endoscopies showed an incidence of EDS of 0.03% [3]. It is often an incidental finding and clinical manifestations are usually nonspecific when they do present. These symptoms include dysphagia, odynophagia, nausea, vomiting, abdominal pain, heartburn, chest pain, and cough [4]. More severe symptoms include hematemesis and obstructive symptoms secondary to the accumulation of casts in the esophageal lumen. It is more common in the elderly with a median age of diagnosis of 65 years and affects both males and females [2].

The pathogenesis is thought to stem from exposures and conditions that cause esophageal insult. This includes physical, chemical, thermal, and immunological damage [1]. Autoimmune conditions that are implicated include celiac disease, pemphigus vulgaris, bullous pemphigoid, and lupus [5,6,7]. Medications involved that are known to cause esophageal irritation include NSAIDs, bisphosphonates, and iron [8,9,10]. Other medications that have been involved are selective serotonin reuptake inhibitor (SSRIs), methotrexate, and clindamycin [2,11,12]. One study showed that 77% of patients with EDS are taking more than 5 medications at the time at diagnosis [4]. They also showed that EDS is associated with chronic debilitation, the most significant feature being a patient that is currently hospitalized.

Many of these findings were evident in our patient. Our patient was hospitalized at the time of diagnosis for ingestion of dye. In addition, she was taking multiple medications. More specifically, our patient was taking SSRIs for major depressive disorder. Multiple case studies have shown an association of EDS with psychoactive agents. One study showed that 73.2% of patients (*n* = 41) were on a psychoactive agent and 51.2% were specifically on an SSRI or SNRI [2]. Another study showed that 65% of patients were on a CNS depressant and 42% were specifically on an SSRI [4]. As early as 1954, reports of EDS having an association with psychogenesis were being discussed [13]. It may be very possible that psychogenesis may predispose patients to developing EDS, or the medications themselves may be contributing to some degree.

EDS is commonly seen in the distal and middle third of the esophagus but can also extend across its entire length [4]. The typical endoscopic findings are vertical sheets of sloughed squamous tissue which appear as multiple columns of strips, described as “gift wrap paper”-like distribution [1]. They can also appear as crumpled, detached mucosa in large fragments and may have associated bleeding [6]. The sloughing membrane is adjacent to intact healthy mucosa and can be easily removed [12]. This can be seen grossly as white plaques or membranes, which are often confused with the white exudates of candida esophagitis. However, in EDS, the white plaques are more diffuse rather than isolated and has a smoother “sloughed” appearance as opposed to the rougher surface of *Candida* [2]. Hart et al. developed three endoscopic criteria for diagnosing EDS. 1: strip(s) of sloughed esophageal mucosa >2 cm in length; 2: normal underlying esophageal mucosa; and 3: lack of ulcerations or friability of immediately adjacent esophageal mucosa.

The histopathologic features of EDS are typically nonspecific. Features that are commonly seen are parakeratosis and intraepithelial splitting [2]. It often creates a two-toned appearance with the deeper squamous mucosa often appearing normal and sometimes completely separated from the superficial layer. Inflammation can range from mild to severe and sometimes is not apparent at all [4].

Prognosis is usually very favorable, and long-term complications related to this entity are very rare. Patients usually have an excellent response to treatment, which entails discontinuation of the offending agent and proton pump inhibitors. Carmack et al. detailed a case study of 12 patients with EDS, all treated with proton pump inhibitors. Of these, 5 underwent follow-up EGD, which showed complete resolution of EDS in 4 patients and mild esophagitis in one [1]. Steroids may be helpful when the inciting factor is autoimmune in nature, such as bullous pemphigoid. Hart et al. demonstrated that 85.7% of EDS patients that underwent follow-up endoscopy showed resolution of the sloughing membranes. Our patient had complete resolution of symptoms after treatment with proton pump inhibitors.

Our patient demonstrated EDS after ingestion of hair dye, the first encounter in current literature. The main toxic component of hair dye is para-phenylenediamine (PPD), an aromatic amine that has been associated with allergic reactions that can present both locally and systemically. Topical exposure causes contact dermatitis characterized by pruritus, erythema, and vesicular or bullous dermatitis in severely affected patients [14]. Uncommon presentations such as lichen planus-like and erythema multiforme-like reactions have also been described [15]. The mechanism has been shown to be driven by inflammatory cytokines such as IL-20 and IL-24 [16]. When ingested, PPD toxicity presents with cervical orofacial angioedema and dysphagia due to local mucosal irritation. This can progress to a systemic allergic reaction that may lead to rhabdomyolysis and acute renal failure with significant morbidity and mortality [17]. Considering the theorized pathogenesis of EDS, it is possible that PPD can cause chemical or immunological damage to the esophageal mucosa causing esophageal sloughing.

Due to the extensive ingredient list in hair dyes, it is difficult to pinpoint a single agent that contributed to our patient’s symptoms. It may have been due to other ingredients or combination of ingredients that are present in the hair dye. Another possible irritant may have been resorcinol. Resorcinol is a moderately toxic and corrosive chemical. There is limited information about its effects, but it has been demonstrated to cause eye, skin, oral, and other gastrointestinal injuries [18].

## 4. Conclusions

Our paper looked at a rare case of esophagitis dissecans superficialis after ingestion of hair dye. EDS is a rarely diagnosed phenomena and has been thought to be caused by exposure to irritants that cause esophageal insult. It is very possible that our patient developed EDS after exposure to hair dye that contains many potential irritants, one of which was para-phenylenediamine. Other studies have looked at a variety of other factors that may contribute to EDS. Our patient was taking a SSRI at the time of diagnosis, which has previously been shown to be associated with EDS. Although this may have been multifactorial in nature, it is very likely that chemical exposure from hair dye caused this case of esophagitis dissecans superficialis.

## Data Availability

No new data were created or analyzed in this study. Data sharing is not applicable to this article.

## References

[1] Carmack S.W., Vemulapalli R., Spechler S.J., Genta R.M. (2009). Esophagitis dissecans superficialis (“sloughing esophagitis”): A clinicopathologic study of 12 cases. Am. J. Surg. Pathol..

[2] Hart P.A., Romano R.C., Moreira R.K., Ravi K., Sweetser S. (2015). Esophagitis Dissecans Superficialis: Clinical, Endoscopic, and Histologic Features. Dig. Dis. Sci..

[3] Fiani E., Guisset F., Fontanges Q., Devière J., Lemmers A. (2017). Esophagitis dissecans superficialis: A case series of 7 patients and review of the literature. Acta Gastroenterol. Belg..

[4] Purdy J.K., Appelman H.D., McKenna B.J. (2012). Sloughing esophagitis is associated with chronic debilitation and medications that injure the esophageal mucosa. Mod. Pathol..

[5] Hage-Nassar G., Rotterdam H., Frank D., Green P.H. (2003). Esophagitis dissecans superficialis associated with celiac disease. Gastrointest. Endosc..

[6] Hokama A., Yamamoto Y., Taira K., Nakamura M., Kobashigawa C., Nakamoto M., Hirata T., Kinjo N., Kinjo F., Takahashi K. (2010). Esophagitis dissecans superficialis and autoimmune bullous dermatoses: A review. World J. Gastrointest. Endosc..

[7] Yogarajah M., Sivasambu B., Jaffe E.A. (2015). Bullous systemic lupus erythematosus associated with esophagitis dissecans superficialis. Case Rep. Rheumatol..

[8] Lamine H., Bochra B., Mouna M., Heykel E., Monia T., Mohamed Masaddak A. (2018). Esophagitis dissecans superficialis due to severe nonsteroidal anti-inflammatory drugs toxicity. Presse Med..

[9] Hokama A., Ihama Y., Nakamoto M., Kinjo N., Kinjo F., Fujita J. (2007). Esophagitis dissecans superficialis associated with bisphosphonates. Endoscopy.

[10] Nasir U.M., Rodgers B., Panchal D., Choi C., Ahmed S., Ahlawat S. (2020). Ferrous Sulfate-Induced Esophageal Injury Leading to Esophagitis Dissecans Superficialis. Case Rep. Gastroenterol..

[11] Abbass K., Haveman L., Gertner E. (2014). Esophagitis dissecans superficialis due to severe methotrexate toxicity. Endoscopy.

[12] Da Silva J.R., Pinho R., Ponte A., Silva M., Furtado A., Carvalho J. (2014). Esophagitis dissecans superficialis associated with severe clindamycin toxicity. J. Gastrointest. Liver Dis..

[13] Mukkanna K.S., Stone N.M., Ingram J.R. (2017). Para-phenylenediamine allergy: Current perspectives on diagnosis and management. J. Asthma Allergy.

[14] Beck R.N. (1954). Oesophagitis dissecans superficialis. Br. Med. J..

[15] Mehta V., Nayak S., Balachandran C. (2010). Pigmented contact cheilitis to paraphenylenediamine. Indian J. Dermatol..

[16] Van Belle A.B., Cochez P.M., de Heusch M., Pointner L., Opsomer R., Raynaud P., Achouri Y., Hendrickx E., Cheou P., Warnier G. (2019). IL-24 contributes to skin inflammation in Para-Phenylenediamine-induced contact hypersensitivity. Sci. Rep..

[17] Umair S.F., Amin I., Urrehman A. (2018). Hair Dye poisoning: “An early intervention”. Pak. J. Med. Sci..

[18] Verma R., Tewari N., Jaiswal S., Rastogi V., Singh D., Tiwari A. (2007). Fatal poisoning caused by oral ingestion of a hair dye. Internet J. Emerg. Intensive Care Med..

